# miRMap: Profiling 14q32 microRNA Expression and DNA Methylation Throughout the Human Vasculature

**DOI:** 10.3389/fcvm.2019.00113

**Published:** 2019-08-08

**Authors:** Eveline A. C. Goossens, Margreet R. de Vries, Karin H. Simons, Hein Putter, Paul H. A. Quax, A. Yaël Nossent

**Affiliations:** ^1^Department of Surgery, Leiden University Medical Center, Leiden, Netherlands; ^2^Einthoven Laboratory for Experimental Medicine, Leiden University Medical Center, Leiden, Netherlands; ^3^Department of Biomedical Data Sciences, Leiden University Medical Center, Leiden, Netherlands; ^4^Department of Laboratory Medicine, Medical University of Vienna, Vienna, Austria; ^5^Department of Internal Medicine II, Medical University of Vienna, Vienna, Austria

**Keywords:** microRNA, DNA methylation, 14q32, DLK1-DIO3, cardiovascular

## Abstract

**Aims:** MicroRNAs are regulators of (patho)physiological functions with tissue-specific expression patterns. However, little is known about inter-vascular differences in microRNA expression between blood vessel types or vascular beds. Differences in microRNA expression could influence cardiovascular pathophysiology at specific sites in the vasculature. Therefore, we aimed to map expression profiles of vasoactive 14q32 microRNAs throughout the human vasculature, as well as expression of vasoactive target genes of the 14q32 microRNAs. Furthermore, we aimed to map the DNA methylation status of the 14q32 locus, which has been linked to cardiovascular disease.

**Methods and Results:** We collected 109 samples from different blood vessels, dissected during general surgery. Expression of a representative set of 17 14q32 microRNAs was measured in each sample. All 17 microRNAs showed a unique expression pattern throughout the vasculature. 14q32 microRNA expression was highest in lower limb vessels and lowest in head and neck vessels. All 17 microRNAs were expressed more abundantly in arteries than in veins. Throughout the human vasculature, we observed trends toward an inverse correlation between expression levels of the 14q32 microRNAs and their vasoactive target genes. DNA methylation of the 3 Differentially Methylated Regions (DMRs) along the 14q32 locus did not associate with primary or mature microRNA expression. However, hyper-methylation in venous coronary artery bypass grafts compared to arterial bypass grafts was observed in the Intergenic-DMR and MEG3-DMR. In patients with end-stage peripheral arterial disease we found differential DNA methylation throughout all DMRs in their lower limb veins. These findings were confirmed in a mouse model for vein-graft disease in which we found regulated 14q32 DNA methylation during the active phase of vascular remodeling. In ischemic tissues of a murine hind limb ischemia model we observed an increase in DNA methylation associated with increased ischemia over time.

**Conclusions:** We show that 14q32 microRNAs are abundantly expressed in the human vasculature and that expression differs significantly between different blood vessels. 14q32 DNA methylation also varies throughout the vasculature and is associated with vascular health, independently of microRNA levels. These findings could have important implications for future research and for future site-specific targeting of epigenetics-based therapeutics.

## Introduction

Cardiovascular disease is the collective term for a variety of diseases that have the highest morbidity and mortality rates worldwide (1). The pathophysiology of cardiovascular disease is multifactorial and different locations in the human vasculature are prone to different types of cardiovascular disease. For example, atherosclerosis occurs predominantly in large and medium sized arteries at locations with disturbed flow, such as bifurcations and curvatures (2), whereas arterial aneurysm formation occurs often in the aortic wall or in intracranial arterial walls (3, 4). This suggests that local differences in the vasculature are important in the pathophysiology of cardiovascular disease. In this study, we focus on microRNAs and differences in their intervascular expression levels.

MicroRNAs (miRs) are a class of small endogenous non-coding RNA molecules of approximately 22 nucleotides in length. Mature microRNAs are single-stranded RNA molecules that bind to the 3′-untranslated region (3′-UTR) of their target mRNAs in order to inhibit mRNA translation into proteins. Individual microRNAs have the ability to inhibit the expression of multiple, up to several hundred, target genes. Therefore, they can function as master switches in (patho)physiological processes (5), including cardiovascular disease. MicroRNAs have been shown to regulate different cardiovascular remodeling processes and pathologies (6–9). Moreover, it was recently found that microRNA expression differs between atherosclerotic plaques and non-atherosclerotic arteries (10–12). MicroRNAs are potentially important new therapeutic targets in the treatment of cardiovascular disease (13–15).

Our group was the first to describe that a large gene cluster of 54 individual microRNA genes on the long arm of human chromosome 14 (14q32) plays a major role in various forms of vascular remodeling and cardiovascular disease. The 14q32 locus is also known as DLK1-DIO3 locus. For example, inhibition of miR-329, miR-494, miR-487b, and miR-495 in mice stimulated neovascularization after hind limb ischemia (16), but inhibition of miR-494 in mice also decreased atherosclerosis and increased plaque stability (17). Moreover, inhibition of miR-495 reduced accelerated atherosclerosis and intimal hyperplasia in mice (18). Furthermore, miR-487b plays a role in angiotensin II-induced aneurysm formation in rats (19). Wang et al. also observed the antiangiogenic effect of miR-329 (20). Moreover, knockdown of miR-494 resulted in an increase in the heart's sensitivity to ischemia-reperfusion injury after myocardial infarction in mice (21). Finally, the 14q32 microRNA cluster was also shown to be associated with bicuspid aortic valve disease (22).

We know that different vascular beds show different susceptibilities to various types of cardiovascular disease and that microRNAs play an important role in cardiovascular pathology (14). Yet, we know little about expression profiles of vasoactive microRNAs throughout the vasculature. MicroRNA expression in general is highly tissue-specific (23), which was already shown by Wienholds et al. (24). However, microRNA expression can also vary significantly between regions within tissues. For example, the vascular microRNA miR-126 has been shown to be vascular endothelial cell-specific (25), whereas another vascular microRNA, miR-145, is mainly expressed in vascular smooth muscle cells (12, 26). Both microRNAs have important roles in neovascularization, atherosclerosis, and aneurysm formation (14, 27–30). 14q32 microRNAs also show differential expression throughout the vasculature. For example miR-329, which is abundantly expressed in the femoral artery of mice, showed low to no expression in the carotid artery and miR-494, on the other hand, is expressed in the carotid artery and is upregulated in unstable human carotid artery lesions (17).

Besides expression of the 14q32 microRNAs, DNA methylation status of the 14q32 locus also plays a role in cardiovascular disease (31). DNA methylation is subject to mutagenic loss, but is conserved in CpG-islands that are predominantly located in promotor regions of genes (32). DNA methylation varies between individuals and, therefore, CpG-rich regions are called Differentially Methylated Regions (DMRs). The human 14q32 locus contains 3 DMRs, namely an Intergenic DMR (IG-DMR), located between the *DLK1* and the *MEG3* genes, a DMR partially overlapping the promotor region of *MEG3* (MEG3-DMR), and a DMR located near the *MEG8* gene (MEG8-DMR), all upstream of the microRNA gene cluster. Variations in the 14q32 DNA methylation status are associated with differences in microRNA expression in type 2 diabetes mellitus (33), murine lupus (34), and oncogenesis (35–39). More importantly, similar to the 14q32 microRNA expression, methylation of the 14q32 locus has been shown to associate with atherosclerosis (31) and with peripheral arterial disease (40).

In this study, we aimed to map expression profiles of 14q32 microRNAs as well as DNA methylation throughout the human vasculature. We collected 109 different blood vessel samples in a biobank that were harvested during general surgery. We profiled the expression of a representative subset of the 14q32 microRNAs and quantified the DNA methylation status within the three 14q32 DMRs. As microRNAs act through expression levels of their target genes, expression levels of confirmed vasoactive target genes for each of the profiled 14q32 microRNAs were measured. We investigated the impact of potential confounding factors on 14q32 microRNA expression and DNA methylation, including age, sex, and malignancies, which have been shown to associate with microRNA expression (41–43) and DNA methylation (35, 36, 38, 39, 44–46) profiles in general. Finally, we confirmed DNA methylation in relevant mouse models of cardiovascular disease i.e., vein graft disease and hind limb ischemia.

## Materials and Methods

### Sample Collection

The miRMap biobank was compiled of 109 different human vascular tissue samples (Supplementary Table 1), which were collected during gastroenterological-, head- and neck-, vascular-, and thoracic surgery performed at the Leiden University Medical Center for various indications. Only surplus vessel tissue was used. Samples were collected in sterile Phosphate Buffered Saline (PBS) (30 mL) with heparin (30 uL of 1,000 units/mL) immediately after dissection and were snap frozen at −80°C after draining of the liquid within 2 h after collection.

All samples were anonymized and data that were collected from patients were only name of vessel, indication of surgery, sex, and age during surgery, so outcomes could not be traced back to individual persons. Collection, storage, and processing of the samples were performed in compliance with the Medical Treatment Contracts Act^1^ and the Code of Conduct for Health Research using Body Material (Good Practice Code, Dutch Federation of Biomedical Scientific Societies, 2002) and the Dutch Personal Data Protection Act (Wet Bescherming Persoonsgegevens, 2001), according to Dutch law for using human tissue rest material in biobanks, following the principles outlined in the Declaration of Helsinki. As we only used surplus, anonymized material, Informed Consent was not required under Dutch law, and therefore not obtained.

Lower limb veins from six patients suffering from peripheral arterial disease in critical ischemic conditions were obtained during lower limb amputation surgery and collected in the Ampubase biobank in the Leiden University Medical Center (Supplementary Table 1). Inclusion criteria for the biobank were minimum age of 18 years and lower limb amputation, excluding ankle, foot, or toe amputations. The exclusion criteria were suspected or confirmed malignancy and inability to give informed consent. Directly after amputation, vessels were dissected from the amputated limb and snap-frozen at −80°C.

Sample collection was approved by the Medical Ethics Committee of the Leiden University Medical Center (Protocol No. P12.265) and written informed consent was obtained from all participants.

### Hind Limb Ischemia Model

All animal experiments were approved by the committee on animal welfare of the Leiden University Medical Center (Leiden, The Netherlands) and all animal procedures were performed conform the guidelines from Directive 2010/63/EU of the European Parliament on the protection of animals used for scientific purposes. Wildtype C57Bl/6 mice (all male, aged 8–12 weeks, housed with water and chow *ad libitum*) were anesthetized by intraperitoneal injection of midazolam (5 mg/kg, Roche Diagnostics), medetomidine (0.5 mg/kg, Orion), and fentanyl (0.05 mg/kg, Janssen Pharmaceuticals). Left hind limb ischemia was induced by a single electrocoagulation of the left femoral arteria proximal to the superficial epigastric artery. Anesthesia was antagonized after surgery with flumazenil (0.5 mg/kg, Fresenius Kabi) and atipamezole (2.5 mg/kg, Orion). Buprenorphine (0.1 mg/kg MSD Animal Health) was provided post-surgery as painkiller. Mice were anesthetized as described above. One group of mice (*n* = 4) was sacrificed without any intervention as baseline control group. Other groups were sacrificed 1 day and 3 days after hind limb surgery (4 mice per group). Hind limb muscles (adductor muscle, gastrocnemius muscle, and soleus muscle) from the ligated left hind limb and its internal control the right hind limb were removed and snap-frozen at −80°C.

### Vein Graft Disease Model

ApoE3^*^Leiden mice, all male, aged 10–20 weeks, were housed with water and a western-type diet (ABdiets, *ad libitum*). This led to hypercholesterolemia above 12 mmol/L, as determined before surgery (Roche Diagnostics). Vein graft surgery was performed by interposition of a donor caval vein in the carotid artery of recipient mice as previously described by De Vries et al. (47). Mice were anesthetized with midazolam (5 mg/kg, Roche Diagnostics), medetomide (0.5 mg/kg, Orion), and fentanyl (0.05 mg/kg, Janssen Pharmaceuticals) and mice were monitored for adequacy of anesthesia by keeping track of breathing frequency and response to toe pinching. After surgery, anesthesia was antagonized with atipamezole (2.5 mg/kg, Orion) and flumazenil (0.5 mg/kg, Fresenius Kabi). As pain relieve buprenorphine (0.1 mg/kg, MSD Animal Health) was provided post-surgery. Mice were sacrificed after 14 or 28 days (7 mice per group) by exsanguination under anesthesia. One mouse of the 14 days group was taken out because humane endpoint criteria were met. Vein grafts and native caval veins were harvested and snap-frozen at −80°C.

### Isolation of Human Umbilical Venous Endothelial Cells (HUVECs), Human Umbilical Arterial Endothelial Cells (HUAECs), Human Umbilical Arterial Smooth Muscle Cells (HUASMCs), and Human Umbilical Arterial Myofibroblasts (HUAFIBs)

Umbilical cords were collected from full-term pregnancies and stored in sterile PBS at 4°C and cell isolation was performed within 7 days. Umbilical cord vascular cells were isolated as previously described by Welten et al. (16). In brief, for HUVEC/HUAEC isolation, a cannula was inserted in the umbilical vein or in one of the umbilical arteries and flushed with sterile PBS. The vessels were infused with 0.075% collagenase type II (Worthington) and incubated at 37°C for 20 min. The collagenase solution was collected and the vessels were flushed with PBS in order to collect all detached endothelial cells. The cell suspensions were centrifuged at 300 g for 5 min and the pellet was resuspended in HUVEC/HUAEC culture medium [M199 (PAA) with 10% heat inactivated human serum (PAA), 10% heat inactivated newborn calf serum (PAA), 1% of penicillin (10,000 U/mL) and 1% of streptomycin (10,000 U/mL) (MP Biomedicals), 150 μg/ml endothelial cell growth factor (kindly provided by Dr. Koolwijk, VU Medical Center, Amsterdam, the Netherlands) and 0.1% (1,000 units/mL) heparin (LEO Pharma)]. HUAECs and HUVECs were cultured in plates coated with 1% gelatin.

The second artery was removed and cleaned from remaining connective tissue. Endothelial cells were removed by gently rolling the artery over a blunted needle. The tunica adventitia and tunica media were separated using surgical forceps. After overnight incubation in HUASMC/HUAFIB culture medium (DMEM GlutaMAX™ (Invitrogen, GIBCO), 10% heat inactivated fetal bovine serum (PAA), 10% heat inactivated human serum, 1% penicillin (10,000 U/mL)/streptomycin (10,000 U/mL) and 1% non-essential amino acids (100X, ref 11140–035) (GIBCO, Life Technologies), both tunicae were incubated separately in a 2 mg/ml collagenase type II solution (Worthington) at 37°C. Cell suspensions were filtered over a 70 μm cell strainer and centrifuged at 300 g for 10 min. Cell pellets were resuspended and plated in culture medium. Cells isolated from the tunica adventitia were washed with culture medium after 90 min to remove slow-adhering non-fibroblast cells.

### Primary Cell Culture

Cells were cultured at 37°C in a humidified 5% CO_2_ environment. Cell-type specific culture medium was refreshed every 2–3 days. Cells were passed at 90–100% (HUVECs, HUAECs, and HUASMCs) or 70–80% confluency (HUAFIBs). HUVECs, HUASMCs, and HUAFIBs were used up to passage six and HUAECs up to passage three. Stocks of isolated HUVECs, HUASMCs, and HUAFIBs up to passage four and HUAECs up to passage two were stored at −180°C in DMEM GlutaMAX™ containing 20% FBS and 10% DMSO (Sigma).

### RNA Isolation

For RNA isolation, frozen human vascular tissues were crushed in liquid nitrogen and total RNA was isolated from tissue powder as well as from cultured cells by standard TRIzol-chloroform extraction, according to the manufacturer's instructions (Thermo Fisher Scientific). RNA concentrations were measured using Nanodrop™ 1000 Spectrophotometer (Thermo Fisher Scientific).

### MicroRNA Quantification

**Figure 1:**
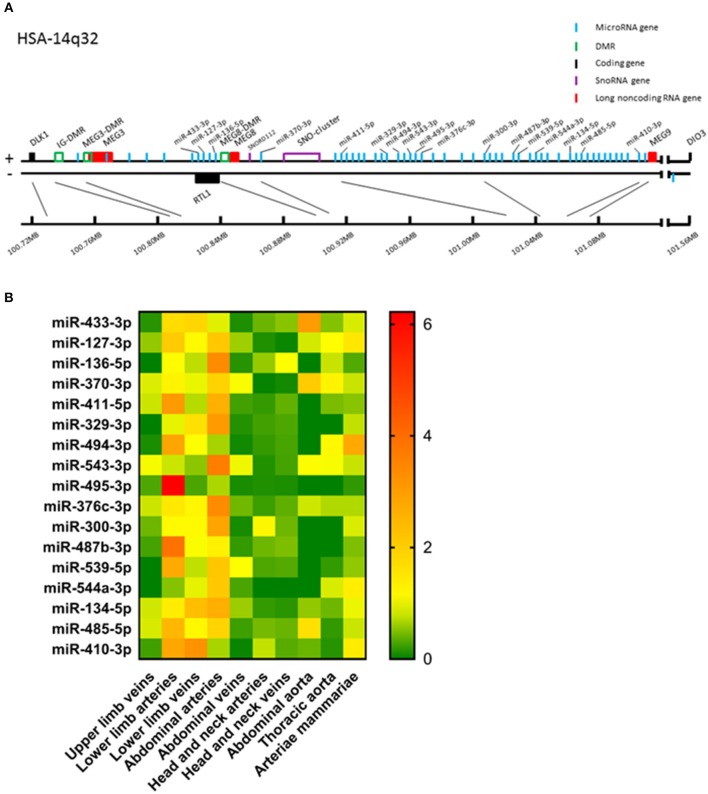
**(A)** Schematic presentation of the human 14q32 locus. Protein coding genes are depicted in black, long non-coding RNA genes in red, measured microRNA genes in blue, DMRs in green, and snoRNA genes in purple. **(B)** Heat map of all analyzed microRNAs. For every microRNA, expression in each vessel group was normalized against the average expression of the microRNA in all samples. Relative expression below average is green, around average is yellow and above average is orange to red. Vessels were divided into the following groups: upper limb veins (*N* = 3), lower limb arteries (*N* = 14), lower limb veins (*N* = 20), abdominal arteries (*N* = 17), abdominal veins (*N* = 6), head and neck arteries (*N* = 9), head and neck veins (*N* = 7), abdominal aorta (*N* = 4), thoracic aorta (*N* = 5), and arteriae mammariae (*N* = 21). MicroRNAs are arranged in the order of chromosomal location of the microRNA gene.

For microRNA quantification, RNA was reversed transcribed using the Taqman™ MicroRNA Reverse Transcription Kit (Thermo Fisher Scientific) and subsequently quantified using microRNA-specific Taqman™ qPCR kits (Thermo Fisher Scientific) on the VIIa7 (Thermo Fisher Scientific). MicroRNA expression was normalized against U6 small nuclear RNA. MicroRNAs analyzed, in order of their genetic location along the 14q32 cluster, were hsa-miR-433-3p (miR-433-3p), hsa-miR-127-3p (miR-127-3p), hsa-miR-136-5p (miR-136-5p), hsa-miR-370-3p (miR-370-3p), hsa-miR-411-5p (miR-411-5p), hsa-miR-329-3p (miR-329-3p), hsa-miR-494-3p (miR-494-3p), hsa-miR-543-3p (miR-543-3p), hsa-miR-495-3p (miR-495-3p), hsa-miR-376c-3p (miR-376c-3p), hsa-miR-300-3p (miR-300-3p), hsa-miR-487b-3p (miR-487b-3p), hsa-miR-539-5p (miR-539-5p), hsa-miR-544a-3p (miR-544a-3p), hsa-miR-134-5p (miR-134-5p), hsa-miR-485-5p (miR-485-5p), hsa-miR-410-3p (miR-410-3p). Of the 54 microRNAs located on the 14q32 locus, we measured the expression of 17 microRNAs in our biobank of human vascular tissue samples. The genes encoding these microRNAs were distributed equally along the locus (Figure 1A) and in a previously published Reverse Target Prediction analysis, these microRNAs were demonstrated to be of potential interest in vascular remodeling (16). Vessels were divided into groups based on location of origin, with each group consisting of samples from at least 3 different individuals. For comparisons between patients with coronary artery disease (CAD) and peripheral artery disease (PAD), we looked both at arteries and veins. For CAD, we included arteriae mammariae and venae saphenae magnae (VSM), both used and prepared for coronary artery bypass grafting, but discarded as surplus length of the bypass. For PAD, we included lower limb arteries and lower limb veins that were either prepared for femoral popliteal bypass grafting, but discarded as surplus length of the bypass, or removed along with amputated tissue after lower limb amputation. Expression levels of microRNAs were calculated relative to U6 expression and expressed as 2^−Δ*Ct*^.

### Primary microRNA Quantification

RNA was reverse transcribed using “high-capacity RNA to cDNA kit” (Thermo Fisher Scientific) and quantified by qPCR using SybrGreen reagents (Qiagen) on the VIIa7. Primary microRNA expression was normalized against U6 and expressed as 2^−Δ*Ct*^. Primary microRNAs and primer sequences are provided in Supplementary Table 4.

### DNA Methylation Assessment

DNA methylation was determined using methylation-sensitive endonucleases, as described previously by Moradi et al. (37). Genomic DNA was isolated using DNeasy Blood & Tissue Kit (Qiagen). Of 109 vessel tissue samples only 78 samples could be used for DNA methylation quantification due to shortage of tissue or to a very low DNA yield (<2 ng/μL). All Ampubase biobank samples had DNA yield > 2 ng/μL. In the murine hind limb ischemia model and in the vein graft model all samples had sufficient DNA yield. After treating the DNA with methylation-sensitive restriction enzymes (Supplementary Table 2; 10 units/reaction according to Manufacturer's Protocol (NEB) with 200 ng DNA per reaction), uncut, and thus methylated, DNA was quantified by qPCR using SybrGreen reagents (Qiagen) on the VIIa7. The estimated methylated DNA fraction (EMF) was expressed relative to an restriction enzyme independent control sequence. Primer sequences and the corresponding CpGs that are potentially cut by methylation-sensitive restriction enzymes are provided in Supplementary Tables 2, 3. Estimated methylation fractions (EMF) were calculated relative to a methylation-independent control, i.e., a stretch of DNA from the same genomic region that did not contain methylation-sensitive restriction sites.

### MicroRNA Targets and DNA Methyltransferases (DNMTs)

For each microRNA, we selected at least one previously confirmed target gene that plays a confirmed role in vascular remodeling. Ninety five out of 109 samples had sufficient RNA yield for mRNA quantification (≥40 ng/μL). mRNA was reverse transcribed using “high-capacity RNA to cDNA kit” (Thermo Fisher Scientific) and quantified by qPCR using SybrGreen reagents on the VIIa7. mRNA expression was normalized against GAPDH and expressed as 2^−Δ*Ct*^. Target mRNAs, DNMTs and primer sequences are provided in Supplementary Table 4.

### Statistical Analyses

Mean values per group are presented ± SEM. Differences between groups were evaluated using Student's *t*-tests with a significance level of α < 0.05. *P*-values were adjusted for multiple testing using Holm-Sidak's method.

A Grubbs' test was used to identify significant outliers (α < 0.05) resulting in exclusion of maximum of one value per data group per microRNA, except for miR-494 in which dataset we excluded three relative expression values in lower leg arteries because of their extremely high value.

Fisher's exact test was performed to evaluate the overall trends toward increases or decreases for all microRNAs between veins and arteries and between non-malignant and peri-malignant vessels.

To evaluate the possible impact of age on expression levels between (primary) microRNA levels and DNA methylation, between DNMT expression and (primary) microRNA expression and between DNMT expression and DNA methylation, linear regression analyses were performed in GraphPad Prism 8.

For the heat map, expression levels of each microRNA were normalized to its own average expression in all samples in the biobank.

One-way ANOVA statistical analysis was performed to detect differences in vascular wall cell types and to assess differences between vessel groups in coronary artery disease (CAD) and peripheral arterial disease (PAD) and for DNA methylation in murine vein graft disease using α < 0.05. To test significance between two groups of the one-way ANOVA analysis, multiple tests were performed and correction for multiple testing with Holm-Sidak's method was done.

To evaluate the possible change of DNA methylation over time, log-transformed, between treated and untreated hind limb muscles within the three DMRs, a mixed model was used in IBM SPSS Statistics 23 (α < 0.05), with time (categorical), muscle, left/right, and DMR as fixed effects, and random intercepts per mouse.

## Results

### Global Expression Patterns of 14q32 microRNAs

Of the 54 microRNA genes located on the 14q32 locus, we measured expression of a selection of a representative subset of 17 microRNAs in our biobank of 109 human vascular tissue samples originating from various locations throughout the human vasculature. These microRNAs were distributed equally along the locus (Figure 1A) and in a previously published Reverse Target Prediction analysis, these microRNAs were demonstrated to be of potential interest in vascular remodeling (16). Expression data were divided into groups based on location of origin of the collected blood vessels. In Figure 1B a heat map of the overall expression of 14q32 microRNAs is presented. Expression of the 14q32 microRNAs was highest in lower limb vessels and abdominal arteries. Some microRNAs, which genes are located next to each other, showed similar expression patterns, such as miR-134-5p and miR-485-5p. However, miR-543-3p and miR-495-3p, also direct genetic neighbors in the cluster, differ in expression pattern. MiR-494-3p and miR-487b-3p, although not located in close proximity from each other within the locus, do show resemblances in relative expression throughout various vessel types. All measured microRNAs, however, showed unique expression patterns, suggesting independent regulation of expression.

### Individual 14q32 microRNA Expression Throughout the Vasculature

**Figure 2:**
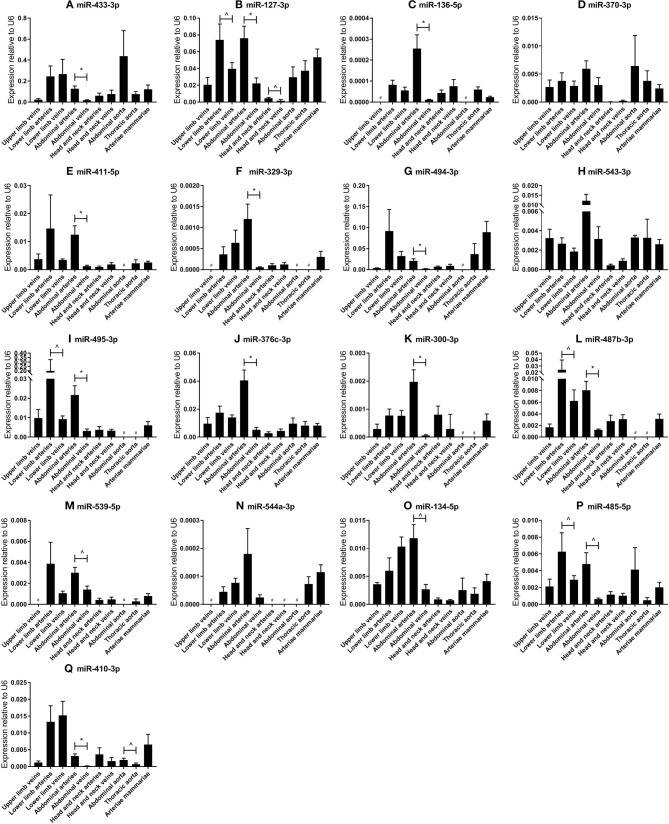
**(A–Q)** MicroRNA expression profiles in human vessel groups. Vessels were divided into the following groups: upper limb veins (*N* = 3), lower limb arteries (*N* = 14), lower limb veins (*N* = 20), abdominal arteries (*N* = 17), abdominal veins (*N* = 6), head and neck arteries (*N* = 9), head and neck veins (*N* = 7), abdominal aorta (*N* = 4), thoracic aorta (*N* = 5), and arteriae mammariae (*N* = 21). Each microRNA shows vessel group specific expression patterns. Mean expression per group is shown. The error bars represent the SEMs. ^∧^*p* < 0.1, ^*^p < 0.05, # <3 samples showed expression and samples were therefore not included in the analyses. Student's *t*-test was used with a significance level of α < 0.05. MicroRNAs are arranged in the order of chromosomal location of the microRNA gene.

Next, we analyzed the relative expression levels of each microRNA in different types of vessels (Figure 2). As presented in Figure 1B, microRNA expression in general was highest in vessel tissues in the lower limbs and the abdomen, and lowest in the head- and neck area. On individual levels, we observed a significant difference in expression between the abdominal arteries and veins for miR-433-3p, miR-127-3p, miR-136-5p, miR-411-5p, miR-329-3p, miR-494-3p, miR-495-3p, miR-376c-3p, miR-300-3p, miR-487b-3p, miR-539-5p, miR-134-5p, miR-485-5p, and miR-410-3p. Similar observations were made for lower limb arteries and veins for miR-127-3p, miR-495-3p, miR-487b-3p, and miR-485-5p. As mentioned above, some microRNAs closely located to each other within the cluster show similar expression profiles, but others vary widely, suggesting a tight and active regulation of expression of each individual microRNA in each different blood vessel. Furthermore, relative expression ranged from <0.0001 in lower limb vessels and abdominal veins (miR-544a-3p, Figure 2N) to 0.4 in abdominal aortas (miR-433-3p, Figure 2A). Among four 14q32 microRNAs, miR-329-3p, miR-487b-3p, miR-494-3p, and miR-495-3p, for which we previously reported a functional role in vascular remodeling (16–19), miR-329-3p showed very low expression, whereas its neighbor in the cluster, miR-494-3p, showed a 100-fold higher expression (Figures 2F,G). Expression levels of these four microRNAs were all below the detection level in abdominal aortic tissue, however they were all harvested during aneurysm repair surgery (Figures 2F,G,I,L). This again implies that microRNA expression is independently regulated.

### Arterial vs. Venous microRNA Expression

**Figure 3:**
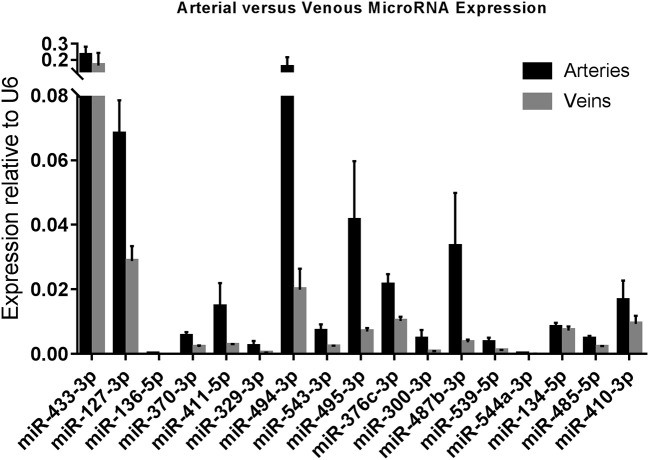
Arterial vs. Venous MicroRNA Expression. Arteries (*N* = 73) vs. Veins (*N* = 36) in miRMap. Mean expression per group is shown. The error bars represent the SEMs. MicroRNAs are arranged in the order of chromosomal location of the microRNA gene.

As we observed differences in expression of 14q32 microRNAs between arteries and veins of the same tissue (e.g., lower limb, abdomen, head, and neck), we divided samples into two groups, namely arteries (*N* = 73) and veins (*N* = 36). Arterial and venous expression of each individual microRNA is depicted in Figure 3. None of the 14q32 microRNAs, individually, showed significant expression differences between arteries and veins after adjusting for multiple testing. However, the overall observation that microRNA expression in arterial samples is higher than in venous samples for each microRNA, was significant (*p* = 0.0027).

### 14q32 microRNA Expression in Vascular Cell Layers of Human Umbilical Cords

**Figure 4:**
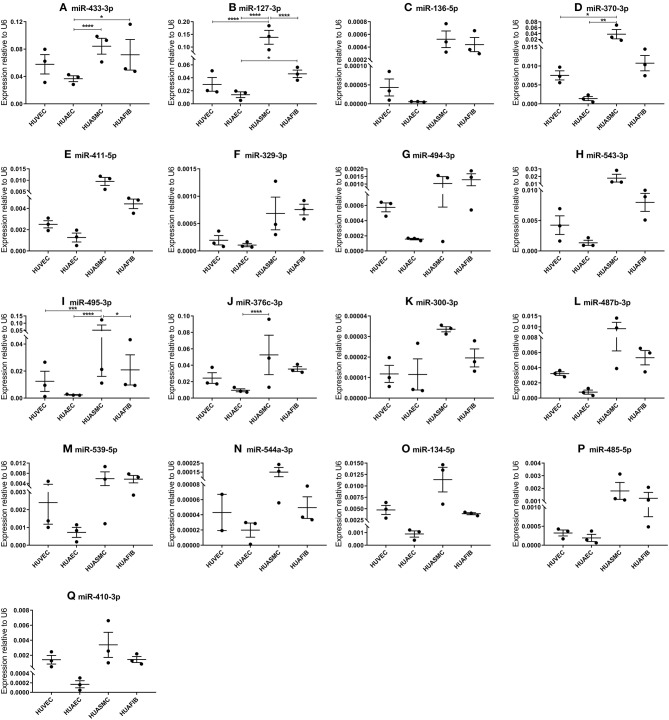
**(A–Q)** MicroRNA expression in human umbilical cord vascular cell layers. Human umbilical venous endothelial cells (HUVEC, *N* = 3), human umbilical arterial endothelial cells (HUAEC, *N* = 3), human umbilical arterial smooth muscle cells (HUASMC, *N* = 3), and human umbilical arterial fibroblasts (HUAFIB, *N* = 3). Mean expression per group is shown. The error bars represent the SEMs. ^*^p < 0.05, ^**^p < 0.01, ^***^p < 0.005, ^****^p < 0.001. One-way ANOVA statistical analysis was performed with a significance level of α < 0.05. MicroRNAs are arranged in the order of chromosomal location of the microRNA gene.

In order to determine in which segment of the vascular wall the 14q32 microRNAs are most abundantly expressed, we measured microRNA expression in cultured primary human umbilical cord cells, realizing of course that the umbilical cord arteries and veins are just two types of blood vessels and that the next findings may differ in other vessel types. We included human umbilical venous endothelial cells (HUVECs), human umbilical arterial endothelial cells (HUAECs), human umbilical arterial smooth muscle cells (HUASMCs), and human umbilical arterial fibroblasts (HUAFIBs). The expression of 14q32 microRNAs in umbilical cord endothelial cells (both venous and arterial) was relatively low compared to arterial smooth muscle cells and arterial fibroblasts. Arterial endothelial cell microRNA expression was also lower than both arterial smooth muscle cell and arterial fibroblast microRNA expression (Figures 4A–Q). We found that HUASMC expression for miR-127-3p was high compared to all other primary vascular cell types (Figure 4B). A similar pattern was found for miR-370-3p and miR-495-3p (Figures 4D,I).

### 14q32 microRNA Expression in Patients With Cardiovascular Disease

**Figure 5:**
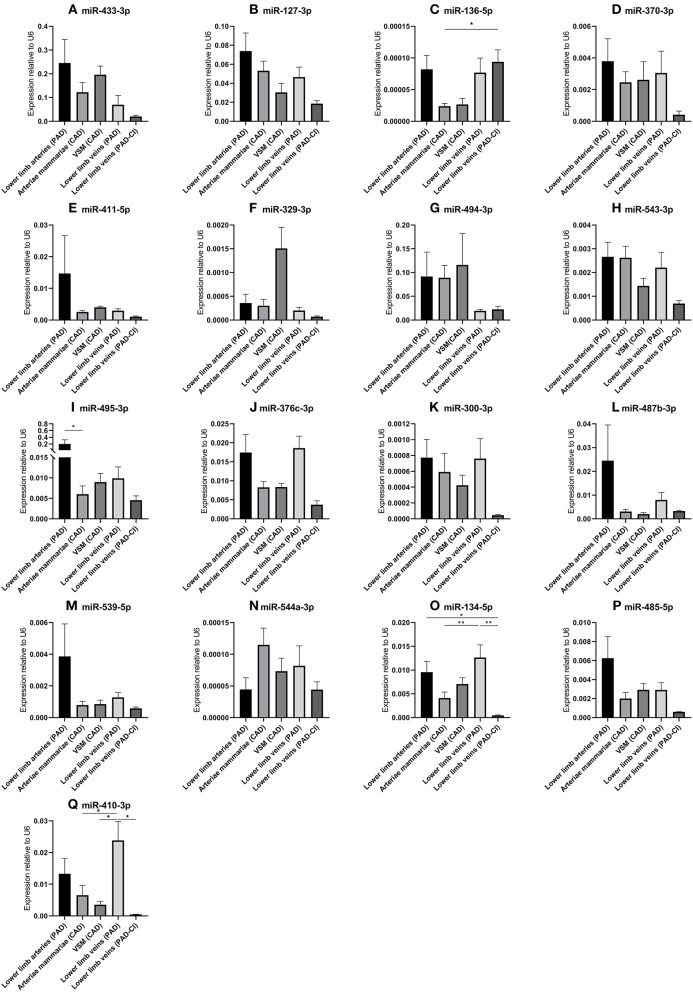
**(A–Q)** MicroRNA expression in human vessel groups of peripheral and coronary artery disease patients. Vessels were divided into the following groups: lower limb arteries from patients with PAD (*N* = 14), arteriae mammariae from patients with CAD (used as arterial graft; *N* = 21), lower limb veins from patients with CAD (used as vein graft; *N* = 8), lower limb veins from patients with PAD (*N* = 12), lower limb veins from patients with critical ischemia (PAD-CI; *N* = 6). Mean expression per group is shown. The error bars represent the SEMs. ^*^p < 0.05, ^**^p < 0.01. One-way ANOVA statistical analysis was performed with multiple testing for differences between all bars individually with a significance level of α < 0.05, corrected for multiple testing. MicroRNAs are arranged in the order of chromosomal location of the microRNA gene.

In order to determine whether the 14q32 microRNAs are also regulated in samples from patients with cardiovascular disease, we selected four groups of blood vessels from the miRMap biobank, harvested during bypass surgery in Coronary Artery Disease (CAD) patients or intermittent Peripheral Artery Disease (PAD) patients. From both CAD and PAD patients, we selected arterial and venous. Furthermore, we selected a group of lower limb veins from patients with PAD in Critical Ischemic conditions (PAD-CI). As shown in Figure 5, except for miR-136-5p and miR-494-3p, microRNA expression is higher in the less ischemic lower limb veins in intermittent PAD compared to the PAD-CI samples.

### 14q32 microRNA Target Gene Expression

**Figure 6:**
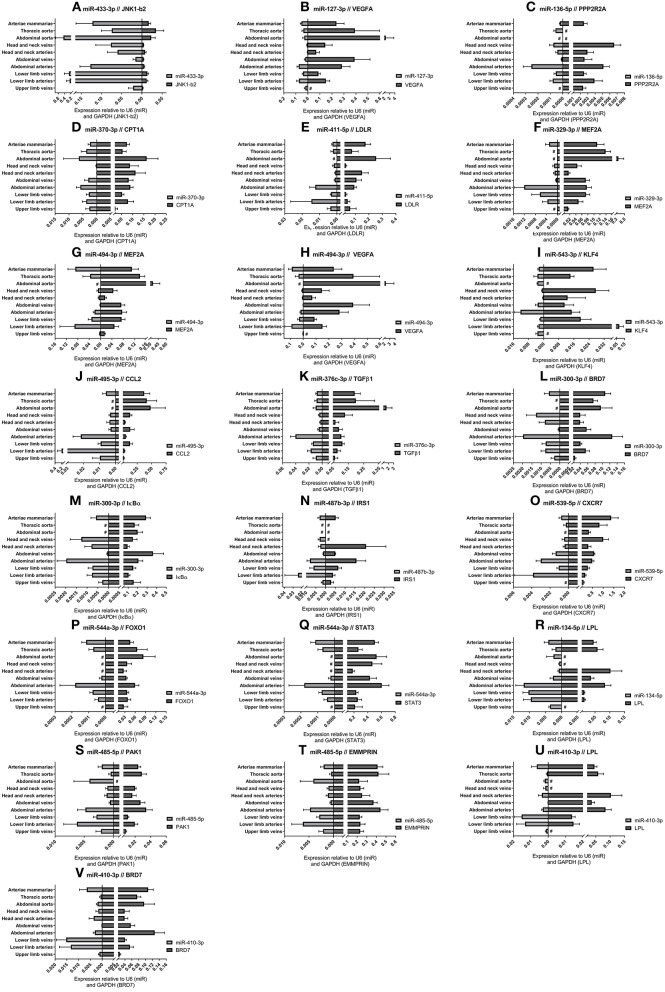
**(A–V)** MicroRNA target genes. Vessels were divided into the following groups: upper limb veins (*N* = 3), lower limb arteries (*N* = 11), lower limb veins (*N* = 18), abdominal arteries (*N* = 14), abdominal veins (*N* = 5), head and neck arteries (*N* = 9), head and neck veins (*N* = 5), abdominal aorta (*N* = 4), thoracic aorta (*N* = 5), and arteriae mammariae (*N* = 18). MicroRNA expression and target gene expression tend to show inversed expression in different vessel groups. Mean expression per group is shown. The error bars represent the SEMs. # <3 samples showed expression and samples were therefore not included in the analyses. MicroRNAs arranged in the order of chromosomal location of the microRNA gene.

For each of the 17 measured 14q32 microRNAs, we selected at least one confirmed target gene with a confirmed vascular function. Vessel groups that show high expression for a specific microRNA, show lower expression of the selected target mRNA and vice versa. This is shown in Figure 6 for the abdominal and thoracic aorta groups, for the arteria mammaria group and for the lower limb vessels. Both high and low microRNA expression show inversed expression of the measured target mRNAs, for example for miR-433-3p, miR-370-3p, and miR-544a-3p (Figures 6A,D,P).

### Methylation of 14q32 DNA Throughout Vasculature

**Figure 7:**
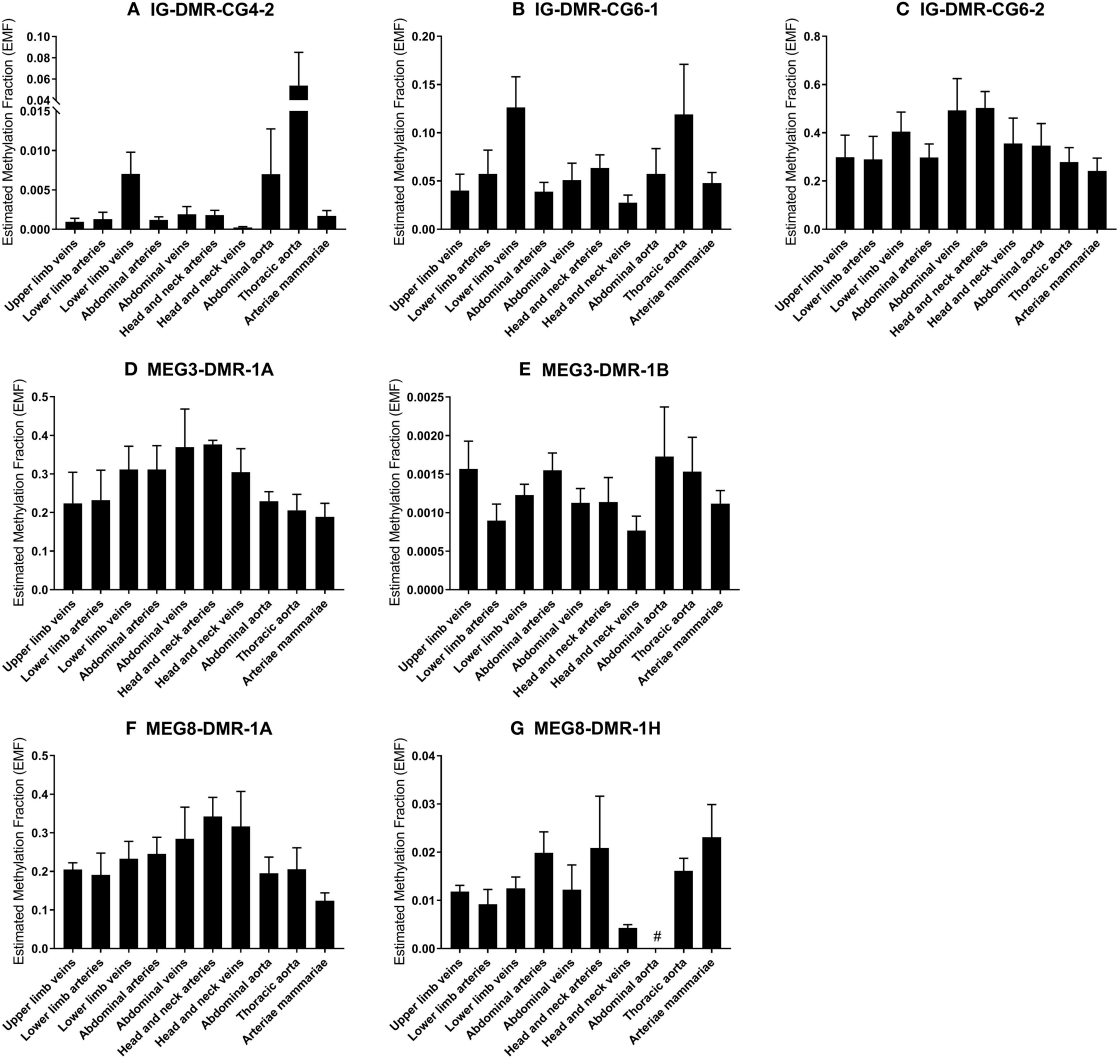
**(A–G)** DNA-methylation at the 3 DMRs along the 14q32 locus in vessel groups. Vessels were divided into the following groups: upper limb veins (*N* = 3), lower limb arteries (*N* = 6), lower limb veins (*N* = 17), abdominal arteries (*N* = 12), abdominal veins (*N* = 4), head and neck arteries (*N* = 5), head and neck veins (*N* = 5), abdominal aorta (*N* = 3), thoracic aorta (*N* = 5), and arteriae mammariae (*N* = 16). Estimated Methylation Fraction (EMF) relative to restriction enzyme-independent control. Mean expression per group is shown. The error bars represent the SEMs. ^#^ <3 samples showed EMF and samples were therefore not included in the analyses.

**Figure 8:**
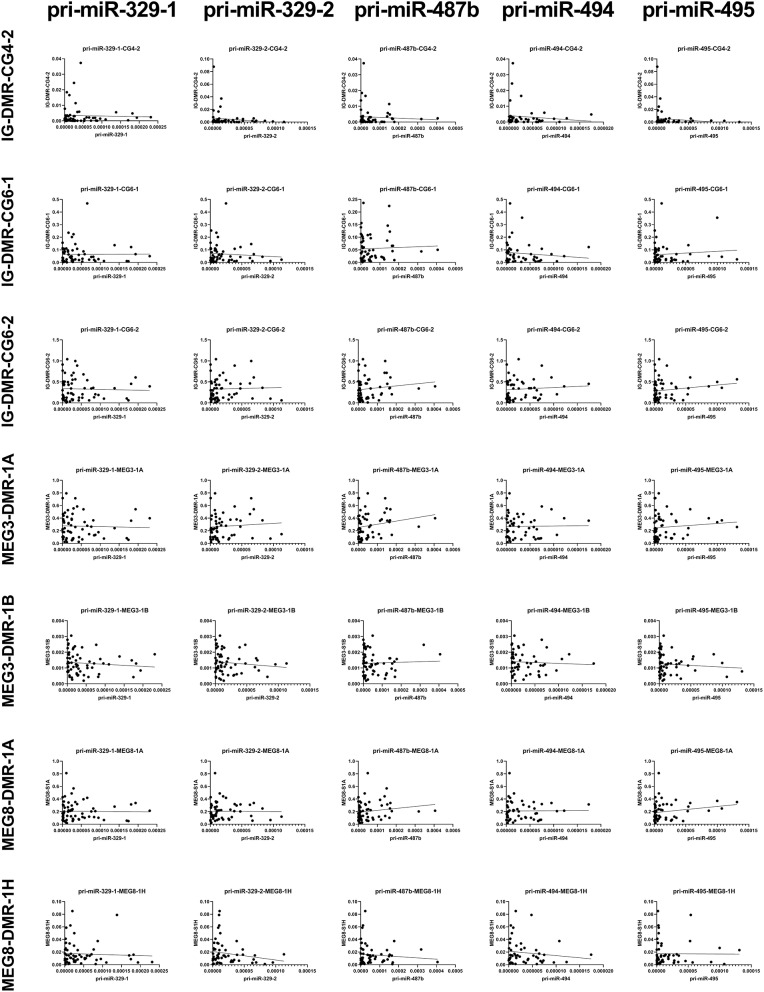
Correlations between primary 14q32 microRNAs and 14q32 DNA methylation. Primary microRNA (pri-miR) expression levels (relative to U6) compared to estimated DNA methylation fraction [EMF (relative to restriction enzyme-independent control)]. Linear regression analyses did not show statistically significant correlations.

We measured the estimated methylation fraction (EMF) of multiple CpGs in the 3 DMRs located along the 14q32 locus (Figure 1A) in 78 miRMap samples. Although we observed distinct patterns of differential methylation throughout the human vasculature for the three different DMRs (Figure 7), we did not observe differences between arteries and veins of the human vasculature. Moreover, we did not find direct correlations between 14q32 EMF and 14q32 microRNA expression individually (data not shown). Since DNA methylation is suggested to mainly affect transcription and the primary form of microRNA is directly affected by transcriptional changes, we also assessed whether there was a correlation between pri-microRNA expression of 4 14q32 microRNAs that are highly regulated in cardiovascular remodeling (miR-329, miR-487b, miR-494, and miR-495) and 14q32 DNA methylation. As shown in Figure 8, none of the pri-microRNAs correlated significantly with EMF of any DMR along the 14q32 locus.

**Figure 9:**
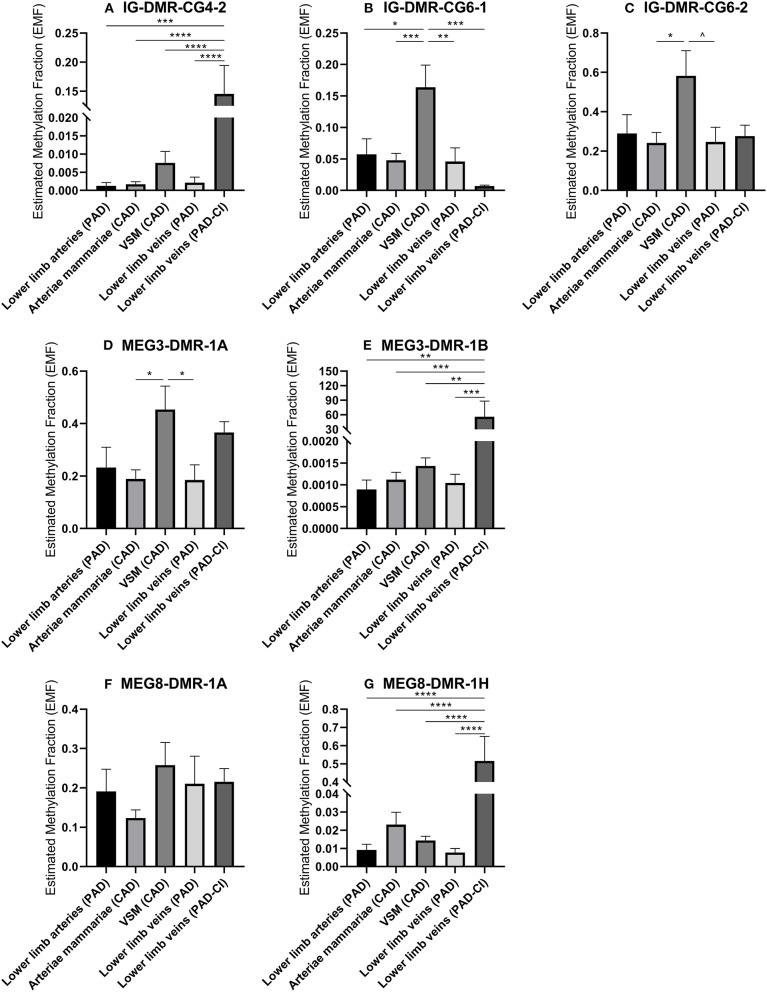
**(A–G)** Methylation of DNA in 14q32 DMRs in human vessel groups of peripheral and coronary artery disease patients. Vessels were divided into the following groups: lower limb arteries from patients with PAD (*N* = 6), arteriae mammariae from patients with CAD (used as arterial graft; *N* = 16), VSMs from patients with CAD (used as vein graft; *N* = 8), lower limb veins from patients with PAD (*N* = 9), lower limb veins from patients with critical ischemia (PAD-CI; *N* = 6). Estimated Methylation Fraction (EMF) relative to restriction enzyme-independent control. Mean expression per group is shown. The error bars represent the SEMs. ^∧^*p* < 0.1, ^*^p < 0.05, ^**^p < 0.01, ^***^p < 0.005, ^****^p < 0.001. One-way ANOVA statistical analysis was performed with multiple testing for differences between all bars individually with a significance level of α < 0.05, corrected for multiple testing.

Changes in DNA methylation of the 14q32 cluster in atherosclerotic lesions were previously reported (31). To further investigate this, we used the previously described four groups of blood vessels from the miRMap biobank, harvested during bypass surgery in CAD or PAD patients, as well as the group of lower limb veins from patients with PAD-CI. We found that in the IG-DMR (IG-DMR-CG6) and in the MEG3-DMR, but not in the MEG8-DMR, venous coronary artery bypass grafts [venae saphenae magnae (VSM)] had a higher EMF than arterial coronary artery bypass grafts (arteriae mammariae; Figures 9B–E). The EMFs did not differ between arterial and venous samples from PAD patients. Furthermore, EMF in venous samples of patients with PAD-CI, collected during amputation, showed a different EMF for each CpG site. EMFs of PAD-CI veins were high compared to PAD veins for IG-DMR-CG4-2, MEG3-DMR-1B, and MEG8-DMR-1H (Figures 9A,E,G), but low in IG-DMR-CG6-1 (Figure 9B). MEG8-DMR-1A did not show any differences between groups (Figure 9F). Together, these data suggest that site specific DNA methylation is linked to cardiovascular disease status directly, rather than indirectly via changes in microRNA expression.

**Figure 10:**
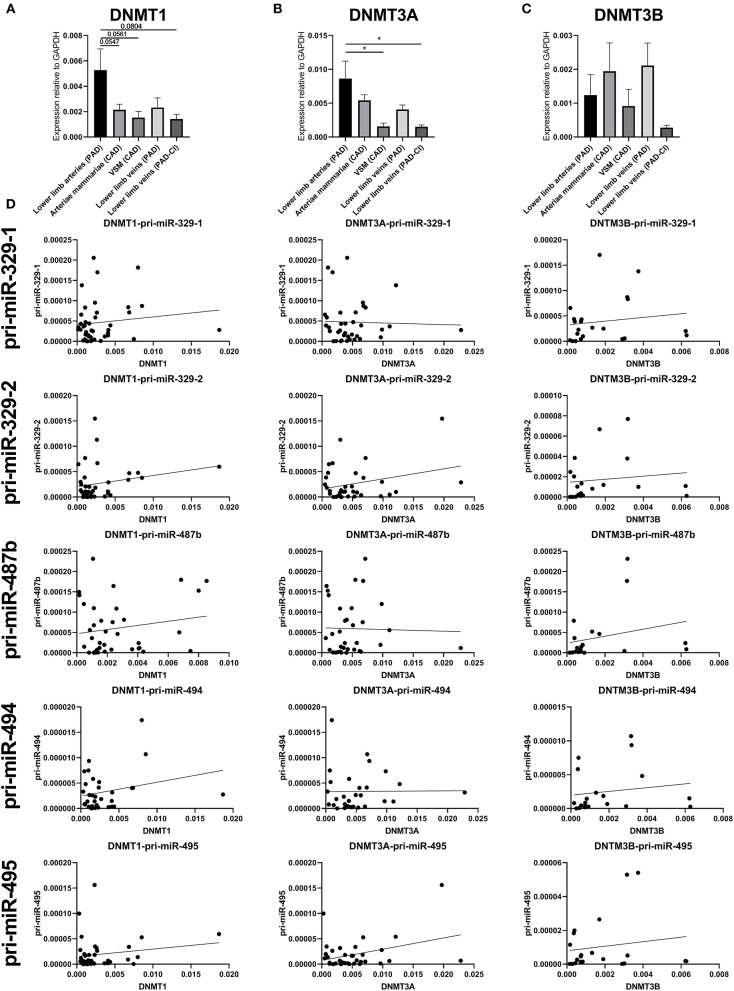
Gene expression of DNMT1, DNMT3A, and DNMT3B and correlations with pri-microRNA expression. Vessels were divided into the following groups: lower limb arteries from patients with PAD (*N* = 11), arteriae mammariae from patients with CAD (used as arterial graft; *N* = 18), VSMs from patients with CAD (used as vein graft; *N* = 8), lower limb veins from patients with PAD (*N* = 10), lower limb veins from patients with critical ischemia (PAD-CI; *N* = 6). The error bars represent the SEMs. ^*^p < 0.05. One-way ANOVA statistical analysis was performed with multiple testing for differences between all bars individually with a significance level of α < 0.05, corrected for multiple testing. Linear regression analyses do not show statistically significant correlations.

To identify whether there is significant correlation between primary microRNA (pri-miR) transcripts and DNA Methyltransferase (DNMT) genes, we measured DNMT gene expression. As we already found that DNA methylation is changed in vascular remodeling processes, we focused on peripheral artery disease (PAD) vascular samples and coronary artery disease (CAD) vascular samples. Figure 10D shows that none of the DNMT genes correlated significantly with pri-microRNAs in miRMap CAD and PAD samples. Mature microRNAs did not associate as well (data not shown). However, it is interesting that DNMT genes expression varies in samples with different cardiovascular diseases, especially between vessels in different stages of PAD. Both DNMT1, which functions in DNA methylation maintenance and DNMT3A, a *de novo* DNA methylation enzyme, are highly expressed in lower limb arteries of PAD patients, show lower expression in lower limb veins of PAD patients and lowest expression is present in lower limb veins of PAD patients with critical ischemia (Figures 10A–C). This implies that DNMT gene expression, like 14q32 DNA methylation, could be linked to cardiovascular disease directly. Furthermore, we assessed whether there is a correlation between DNA methylation and DNMT gene expression (Supplementary Figure 1). A significant correlation could only be found between DNMT3A and MEG3-DMR-1A (*p* = 0.03).

### 14q32 DNA Methylation in Murine Models for Cardiovascular Disease

**Figure 11:**
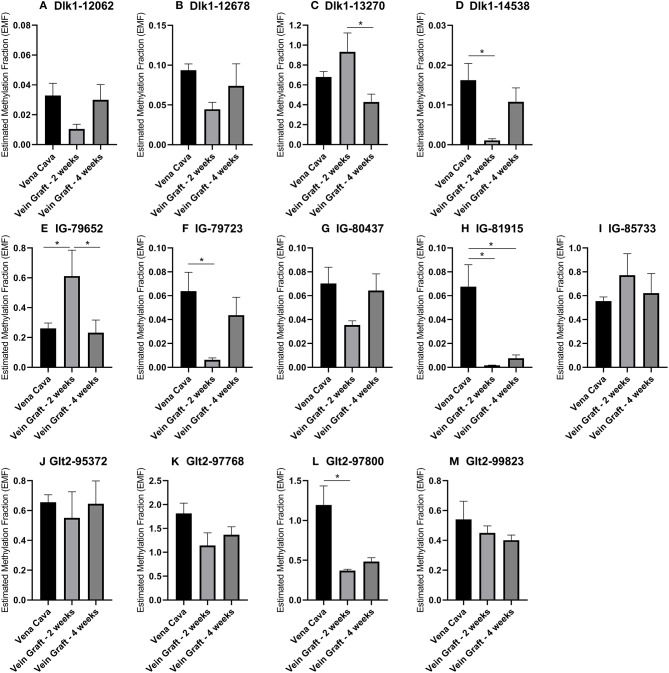
**(A–M)** 14q32 DNA methylation in murine vein graft disease. Native venae cavae (*N* = 13) were compared to venae cavae used as vein grafts, harvested either after 2 weeks (*N* = 6) and after 4 weeks (*N* = 7) in the different CpG-positions along the genome. At 2 weeks after surgery, Estimated Methylation Fraction (EMF) was up- or downregulated and at 4 weeks after surgery this change seemed to go back to the EMF of the native venae cavae. EMF is expressed relative to restriction enzyme-independent control. Mean expression per group is shown. The error bars represent the SEMs. ^*^p < 0.05. One-way ANOVA statistical analysis was performed with multiple testing for differences between all bars individually with a significance level of α < 0.05, corrected for multiple testing.

To confirm the associations of 14q32 DNA methylation with both vein graft disease and peripheral artery disease, we used two different mouse models. In a murine model for vein graft disease, we found that 14q32 DNA methylation was changed during the active phase of vascular remodeling, i.e., at 2 weeks after vein graft surgery (Figure 11). After 28 days a more stable phase of vein graft disease (48, 49), EMF seems to go back toward the EMF of the native vena cava (Figure 11). DNA methylation, however, was not shown to increase or decrease DMR-specifically, as we found both EMF increases and EMF decreases at 2 weeks after vein graft surgery within one DMR, namely IG-DMR (Figures 11E,F).

**Figure 12:**
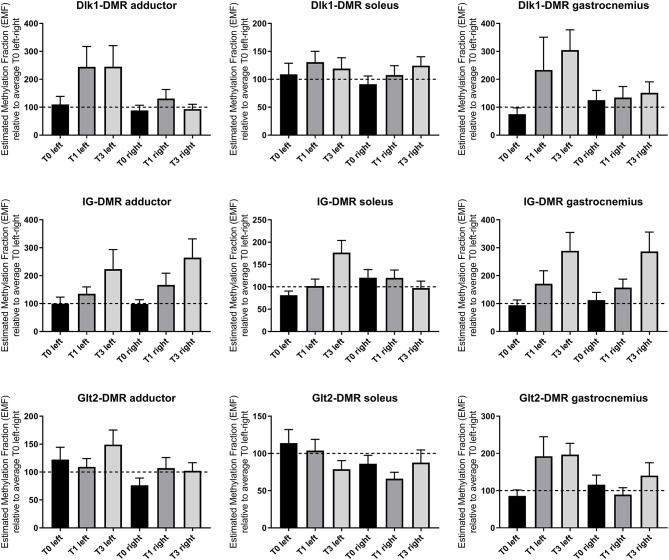
14q32 DNA methylation in murine hind limb ischemia model. Hind limb muscles (adductor, soleus and gastrocnemius) harvested before (*N* = 4), 1 day after (*N* = 4), and 3 days after left femoral artery ligation (*N* = 4). Muscles from the right paw were used as internal control. Estimated Methylation Fraction (EMF) relative to restriction enzyme-independent control is expressed relative to the average EMF of T0 in left and right (set to 100% and indicated as the dotted line). Mean expression per group is shown. The error bars represent the SEMs. On all data together, a Mixed Model is performed and showed a significant change in DNA methylation over time in ischemic muscle tissues in the three DMRs (*p* = 0.009).

Hind limb ischemia was induced via femoral artery ligation, to mimic PAD in mice. We have previously shown a large scale upregulation for 14q32 microRNAs in response to hind limb ischemia in mice (16). Here, we measured 14q32 DNA methylation of the three DMRs (Dlk1-DMR, IG-DMR, and Glt2-DMR) in three different muscles (adductor muscle, gastrocnemius muscle, and soleus muscle) at three different time points (Figure 12). Using a Mixed Model in SPSS, on all data together, we found a significant change in DNA methylation over time in ischemic muscle tissues in the three DMRs (*p* = 0.009).

Together, these findings indicate that associations between 14q32 microRNA expression and cardiovascular disease as well as between DNA methylation and cardiovascular disease are conserved over species.

### Potential Confounding Factors

As microRNA expression and DNA methylation can be influenced by different factors, we assessed the influence of the most common confounding factors: sex, age, and malignancy. In the miRMap biobank, neither 14q32 microRNA expression nor DNA methylation differed between men and women. Furthermore, no correlation with age was observed (data not shown). Finally, we selected vessels resected from locations near resected tumors (abdominal and head and neck tumors) and compared expression with vessels originating from these same locations from patients without malignancies (Supplementary Figure 2A). Although we did not observe significant differences for individual microRNAs, of the 17 measured microRNAs, 16 microRNAs showed higher expression in the peri-malignancy samples (*p* = 0.0167). For DNA methylation, no differences in EMF nor a trend toward hypo- or hypermethylation in any of the groups was observed (Supplementary Figure 2B).

## Discussion

All 14q32 microRNAs measured in this study are expressed in the human vasculature, but not all microRNAs are expressed in all vessel types. In fact, each 14q32 microRNA had its own unique expression “fingerprint” throughout the vasculature. Expression levels of the 14q32 microRNAs varied widely in each type of vessel. These findings confirm the existence of tight individual regulation of 14q32 microRNA expression which should be taken into account in future studies. Moreover, our study demonstrates the complexity of human microRNA expression patterns, which has been studied extensively in different blood components (50, 51), but not in the vessel wall itself. This may have implications for future microRNA-based therapies, either as a limitation, if the target microRNA is not expressed at a vascular target site, or as a potential advantage when highly specific vessel type microRNA expression allows us to modulate microRNA function locally, avoiding systemic vascular side effects (14). DNA methylation of the 14q32 locus also varied over different blood vessels, but was not directly associated with microRNA expression, as was claimed by previous reports (31, 35). Our results underline the complex transcriptional landscape of non-coding RNAs in general.

Interestingly, we observed the highest expression in lower limb vascular tissues and lowest expression in head- and neck vascular tissues was clearly observed. Both vessel groups originate from locations prone to atherosclerosis. However, even though the pathophysiologies of both carotid artery disease and peripheral artery disease are consistent (2) and affected by the same risk factors (52), we found differential expression of 14q32 microRNAs, which have previously been shown to associate with atherosclerotic disease (17).

The observation that all measured 14q32 microRNAs had higher expression in arteries than in veins can be explained by a previous finding that 14q32 microRNAs are expressed abundantly in fibroblasts, which are highly present in the adventitial layer of arteries (16, 19). This was confirmed by the high arterial fibroblast 14q32 microRNA expression. Furthermore, it was observed that arterial smooth muscle cells also showed high expression. This was reported previously for a non-14q32 microRNA, miR-195 (53). This microRNA showed more prominent expression in human aortic smooth muscle cells than in HUVECs (53), however, this could also be an effect of the vascular bed, rather than the cell type. Although vascular SMCs and adventitial fibroblasts are mainly associated with arteries, they are also present in veins, albeit less prominent. Our finding that 14q32 microRNA expression is higher in both HUASMCs and HUAFIBs and low in both HUVECs and HUAECs, suggests that these vascular wall cell layers are the major contributors to 14q32 microRNA expression in the vascular wall, at least in umbilical cord vessels.

The fact that we observed microRNA-specific expression fingerprints implies individual regulation of 14q32 microRNA expression. MicroRNA expression can be regulated by many different factors, both acting during transcription and during post-transcriptional processing. RNA Binding Proteins (RBPs) for example, are post-transcriptional regulators of microRNA expression. RBPs are able to bind precursor microRNAs and promote or inhibit microRNA maturation. Thereby, RBPs directly influence microRNA expression. Myocyte Enhancer Factor 2A (MEF2A) is such an RBP, which regulates post-transcriptional regulation of both miR-329 and miR-494 (54), but Cold-Inducible RNA-Binding Protein (CIRBP) and Hydroxyacyl-CoA DeHydrogenase trifunctional multienzyme complex subunit Beta (HADHB) are also RBPs that regulate 14q32 microRNAs miR-329 and miR-495 (55). However, even between these microRNAs, differences in expression levels and patterns were found. This suggests that MEF2A, CIRBP, and HADHB are only a small part of the complex regulation of microRNA expression. As already suggested by Treiber et al., the interaction between RBPs and microRNAs is a complex mechanism (56) and further studies are needed to elucidate the full mechanism of and interplay between transcriptional and post-transcriptional factors that affect localized microRNA expression.

Expression of target genes of 14q32 microRNAs was also assessed. In a previous study MEF2A was identified as direct target of miR-329 (16). Here, we found that lower expression of miR-329-3p, as well as miR-494-3p, corresponds with higher expression of MEF2A and vice versa. VEGFA is a confirmed target of miR-494 (16), as well as of miR-127 (57), and we found that expression of these microRNAs is indeed inversely correlated with expression of VEGFA throughout the vasculature. MiR-495 targets CCL2 and thereby affects proliferation and apoptosis of HUVECs (58). In the vessel where CCL2 is highest expressed, the aorta, miR-495-3p was not expressed. We show here that miR-433-3p is highly expressed in the human vasculature. It has been shown previously that miR-433 is also abundantly expressed in cardiac fibrosis (59) and that the miR-433 target gene JNK1-b2, shows decreased expression in cardiac fibrosis. We confirmed the inversed relation between miR-433 and JNK1-b2 in miRMap. MiR-370 has been described to directly inhibit CPT1A expression and thereby reduce fatty acid β oxidation in lipid metabolism (60). We also found an inversed correlation between miR-370 expression and CPT1A expression. FOXO1 has been described to be a direct target of miR-544 in colorectal cancer development (61). FOXO1 itself has been found to inhibit endothelial growth and proliferation (62) and wound closure and vascular density was reduced upon deletion of FOXO1 (63). Again, we observed an inverse relationship between miR-544 and FOXO1 expression profiles. Therefore, we could conclude that the measured 14q32 microRNAs act as regulators of their target mRNAs in vascular remodeling.

DNA methylation in the 14q32 locus is associated with tissue specificity in oncogenesis (64), but it is also associated to several (patho)physiological conditions in cardiovascular diseases like atherosclerosis (31) and cardiac fibrosis (65). Therefore, we quantified DNA methylation of multiple CpGs in the three different DMRs along the 14q32 locus in order to assess a possible relation between 14q32 DNA methylation and the different vascular origins. Differential DNA methylation was measured, but we did not find clear patterns overall throughout the human vasculature. Although it had previously been shown that hypo-methylation of the 14q32 DMRs is associated with increased 14q32 microRNA expression and vice versa (31, 35), we did not find a direct correlation between 14q32 DNA methylation and 14q32 microRNA expression in miRMap. Moreover, DNA methylation did not correlate with pri-microRNA expression. The lack of direct associations between DNA methylation and microRNA expression indicates that DNA methylation can have other functions than merely regulation of transcription. An example of such “novel” functions was published by Shayevitch et al., demonstrating that DNA methylation can function as regulator of alternative splicing of mRNA (66). This suggests that transcription and interaction between epigenetic features is a much more complex mechanism than has been thought and such alternative functions of the epigenome require further in-depth investigations in the future.

In patients with CAD who underwent coronary artery bypass grafting, significantly higher 14q32 DNA methylation of two DMRs was found in pre-implantation venous bypass grafts than in pre-implantation arterial bypass grafts. We know that coronary artery bypass graft patency is still significantly higher in arterial than in venous bypass grafts (67–69). Future research into the effect of 14q32 DNA methylation on graft patency is needed to assess a direct relationship. Furthermore, we found a highly genomic location-dependent DNA methylation status of critical ischemic lower limb veins compared to intermittent ischemic lower limb veins. 14q32 DNA methylation status is associated with atherosclerosis (31) and ischemic arterial disease (40). The observed differential DNA methylation statuses imply that DNA methylation itself is a disease dependent mechanism of gene expression regulation, independent of microRNA expression. Furthermore, it is interesting that DNMT gene expression varies in samples with different cardiovascular diseases, especially between vessels in different stages of PAD. Both DNMT1, which functions in DNA methylation maintenance, and DNMT3A, a *de novo* DNA methylation enzyme, are highly expressed in lower limb arteries of PAD patients and lowest expression is present in lower limb veins of PAD patients with critical ischemia. This implies that DNMT gene expression, like 14q32 DNA methylation could be linked to cardiovascular disease directly. A significant correlation between DNMT gene expression and 14q32 DNA methylation could only be found between DNMT3A and MEG3-DMR-1A. The absence of other significant correlations within this analyses could be explained by the fact that we have measured the status of DNA methylation and RNA expression at one timepoint, whereas DNA methylation is a dynamic process and we only measured in a static situation. Future research is needed to fully elucidate this independent mechanism and the interplay between DNA methylation and cardiovascular disease.

Conservation between the human 14q32 locus and the murine 12F1 locus has been described previously (23). In murine models for PAD and vein graft disease, we found increasing DNA methylation over time after hind limb ischemia. Furthermore, we observed changes in DNA methylation at 2 weeks after vein grafting, when vascular remodeling is most active vs. a normalization of the DNA methylation status at 4 weeks after vein grafting. At 4 weeks, the remodeling process of the vein graft model slows down toward a new stable state (48, 49). This suggests that 14q32 DNA methylation status is actively regulated during vascular remodeling. Unfortunately, we have no way to determine the exact stage of remodeling in the human atherosclerotic vascular samples that we obtained. However, the findings that DNA methylation directly associates with cardiovascular disease in the human vasculature, was fully supported by our murine models.

Confounding factors that the miRMap study design allowed us to include, namely sex, age, and malignancy, and that are known to affect development and progression of cardiovascular diseases (41–43), were assessed. We did not find a correlation of age and sex with 14q32 microRNA expression as well as with 14q32 DNA methylation. However, in 16 out of 17 14q32 microRNAs, expression in peri-malignancy vessels was higher than in non-malignant control vessels, indicating that malignancies do influence 14q32 microRNA expression in blood vessels in their vicinity. On the other hand, 14q32 DNA methylation did not differ between vessels closely located to malignancies and their control vascular tissues, unlike what has been described in literature for several types of cancer (35, 36, 39). However, this was in the malignancy itself and not in vascular tissue nearby.

In conclusion, 14q32 microRNAs showed to be highly diverse in expression pattern throughout the human vasculature. In this study, we focused on the 14q32 microRNA cluster, but it is likely that other vasoactive microRNAs outside the 14q32 cluster, like the endothelial-specific microRNA miR-126 and the smooth muscle cell-specific microRNA miR-145, show specific vascular fingerprints as well. Furthermore, although DNA methylation could not be linked to microRNA expression, it is directly linked to both cardiovascular disease status and vessel location. Vessel-specific microRNA expression and DNA methylation profiles could impact both the development and treatment of cardiovascular disease, merit more attention in future studies into the mechanistic role of microRNAs and DNA methylation in cardiovascular pathology and should be taken into account in treatment of cardiovascular diseases via microRNAs.

## Data Availability

Datasets are available on request to the corresponding author.

## Ethics Statement

Cohort 1 - miRMAP: All samples were anonymized and data that were collected from patients were only name of vessel, indication of surgery, sex, and age during surgery, so outcomes could not be traced back to individual persons. Collection, storage and processing of the samples were performed in compliance with the Medical Treatment Contracts Act (WGBO, 1995) and the Code of Conduct for Health Research using Body Material (Good Practice Code, Dutch Federation of Biomedical Scientific Societies, 2002) and the Dutch Personal Data Protection Act (WBP, 2001), according to Dutch law for using human tissue rest material in biobanks, following the principles outlined in the Declaration of Helsinki. As we only used surplus, anonymized material, Informed Consent was not required under Dutch law, and therefore not obtained.

Cohort 2 - Ampubase: Sample collection was approved by the Medical Ethics Committee of the Leiden University Medical Center (Protocol No. P12.265) and written informed consent was obtained from all participants.

## Author Contributions

EG collected the miRMap biobank samples, performed the experiments, analyzed the data, and wrote the manuscript. MV performed the experiments and edited the manuscript. KS collected the Ampubase biobank samples and edited the manuscript. HP performed analyses and edited the manuscript. PQ and AN designed and supervised the experiments and wrote the manuscript. All authors contributed to manuscript revision, read and approved the submitted version.

### Conflict of Interest Statement

The authors declare that the research was conducted in the absence of any commercial or financial relationships that could be construed as a potential conflict of interest.

## Footnotes

^1^Wet op de Geneeskundige Behandelingsovereenkomst (WGBO), artt. 7:446-468 Burgerlijk Wetboek, 1995.
