# Supplementary material for: miRMap: Profiling 14q32 microRNA Expression and DNA Methylation Throughout the Human Vasculature
Source: Front Cardiovasc Med. 2019 Aug 8;6:113. doi: 10.3389/fcvm.2019.00113 (PMC6694280; doi:10.3389/fcvm.2019.00113)
Supplement: Supplementary file 1 [file Data_Sheet_1.docx]

**miRMap: Profiling 14q32 microRNA Expression and DNA Methylation Throughout the Human Vasculature**

Eveline A.C. Goossens, Margreet R. de Vries, Karin H. Simons, Hein Putter, Paul H.A. Quax, A. Yaël Nossent

**Supplementary Data**

**Supplementary Table 1.** Vascular tissue samples used for microRNA analysis

| **miRMap biobank** | **N** | **Mean age (SD)** | **Male (%)** | **Peri-malignancy (%)** |
| --- | --- | --- | --- | --- |
| *Upper limb veins* | 3 | 51.3 (12.1) | 100 | 0 |
| *Lower limb arteries* | 14 | 60.6 (15.1) | 71.4 | 0 |
| *Lower limb veins* | 20 | 69.1 (13.1) | 65.0 | 0 |
| *Abdominal arteries* | 17 | 65.4 (11.6) | 70.6 | 64.7 |
| *Abdominal veins* | 6 | 58.3 (15.7) | 83.3 | 66.7 |
| *Head and neck arteries* | 9 | 54.0 (28.0) | 33.3 | 22.2 |
| *Head and neck veins* | 7 | 50.7 (21.5) | 57.1 | 28.6 |
| *Abdominal aorta* | 4 | 65.8 (14.1) | 25.0 | 0 |
| *Thoracic aorta* | 5 | 69.8 (16.7) | 40.0 | 0 |
| *Arteriae mammariae* | 21 | 64.2 (10.0) | 66.7 | 0 |
| *Atrial appendage* | 1 | 50 (-) | 100 | 0 |
| *Radial artery* | 1 | 42 (-) | 100 | 0 |
| *Umbilical artery* | 1 | 0 (-) | unknown | 0 |
| **Total** | 109 | 61.4 (17.1) | 63.3 | 17.4 |
|  |  |  |  |  |
| **Ampubase biobank** | **N** | **Mean age (SD)** | **Male (%)** | **Peri-malignancy (%)** |
| *Lower limb veins* | 6 | 61.6 (17.4) | 100 | 0 |

**Supplementary Table 2.** Methylation-sensitive restriction enzymes used for DNA-methylation quantification.

| **Restriction Enzyme** | **Cut site** |
| --- | --- |
| AciI | C\|CGC |
| HhaI | GCG\|C |
| BstUI | CG\|CG |
| HpaII | C\|CGG |

**Supplementary Table 3.** Methylation sites and corresponding qPCR primer sequences and methylation-sensitive restriction enzymes.

| **Name of methylation site** | **CpG positions in genome (GRCh38/hg38)** | **Sequence Forward Primer** | **Sequence Reverse Primer** | **Enzyme** |
| --- | --- | --- | --- | --- |
| IG-DMR-CG4-2 | 100809537-8 | CTTCCTGGCCCTTCCCTG | TGATAACCTGCGGATCTGAGA | AciI |
| IG-DMR-CG6-1 | 100810930-1 100810932-3 100810948-9 | GGATCTGTGAGAAATGACTTCG | CAGGCACCTGGTGAGTCG | AciI |
| IG-DMR-CG6-2 | 100811108-9 100811115-6 | CTAATTGCCAGCGATTTGC | TCCATTGTGGCCAGTTACAG | AciI |
| DMR-MEG3-A | 100825889-90  100825914-5  100825929-30 | CACGCAGGGAAAAAGCAC | ACCCAGATTGCAGCAAAGAA | AciI |
| DMR-MEG3-B | 100825889-90  100825891-2 |  |  | BstUI |
| DMR-MEG8-1A | 100904620-1 | CCTGGGCCACATTTCTGAG | CGCATCTGTCGCAGTTAAAA | AciI |
| DMR-MEG8-1H | 100904603-4 100904623-4 |  |  | HhaI |
| DMR-Control |  | TTGCTACAAAGGATCTGAGCTG | GTTGCAAACCAGGGTGAAGT | - |
|  |  |  |  |  |
| **Name of methylation site** | **CpG positions in genome (AJ320506.1)** | **Sequence Forward Primer** | **Sequence Reverse Primer** | **Enzyme** |
| Dlk1-DMR | 12062 | AGCTGCAATGCTCATTCCTAGTG | TAGTGGTCTATTCACCCAAGTGC | HpaII |
|  | 12678 | GGAAAGGGCATGGGAGAGGAC | CCATCGTTCTCGCATGGGTTAGG | HhaI |
|  | 13270 | AGGCCATCTGCTTCACCATCC | CGCTGTTATACTGCAACAGGAG | HhaI |
|  | 14538 | GCCCAAGACTCCACCTCATGC | CACCCCACAAGCCATAGTGTC | HpaII |
| IG-DMR | 79652 | GGATCCTGACCTATGTGTACCTCTG | ACGGACCGTGTGTATGTGCTGTAG | HhaI |
|  | 79723 |  |  | HpaII |
|  | 80437 | TCTTGTGGCAAAGGTACGTGACTG | GTATGCTATGCATTTGTGCTGAAGG | HpaII |
|  | 81915 | AGCTGACTTCCTTCAGCCACAGT | TTGACCCTGTGAGAGATGCTCAG | HpaII |
|  | 85733 | AGTTTCTGGGAAACGTACAGAAGG | CACTTCTCTGCAAGGCCAAGTC | HhaI |
| Glt2-DMR | 95372 | GGTCGGGAGCGAGATGGGTTG | GCGTCCATGACACCCTAAATCAC | HhaI |
|  | 97768  97800 | TCCCGTTCATGGCTCATGTGTCTC | GCCCTGGAAATGACCGCACACTC | HhaI  HpaII |
|  | 99823 | CCCTCTCAGTTTCCCAAACCTG | CCAAGGTATCCTGGAAGAGCTGAC | HpaII |
| DMR-Control |  | GGTGCCAGCAGAGACTTACACAG | CATGCCCTTTGACACTTAGTATGC | - |

**Supplementary Table 4.** qPCR primer sequences for target mRNAs, pri-miRs and DNMTs

| **Primer** | **Sequence Forward Primer** | **Sequence Reverse Primer** | **Targeted by microRNA** |
| --- | --- | --- | --- |
| HSA-CXCR7 | ACAGCACAGCCAGGAAGG | TCCCTGGCTCTGAGTAGTCG | 539 |
| HSA-TGFβ1 | GGCTACCATGCCAACTTCTG | CCGGGTTATGCTGGTTGTA | 376c |
| HSA-KLF4 | AGGGAGAAGACACTGCGTCA | ACGATCGTCTTCCCCTCTTT | 543 |
| HSA-JNK1-B2 | AGAATCAGACTCATGCCAAGC | GGGATTTCTGTGGTGTGAAAA | 433 |
| HSA-CPT1a | ATCAAGAAATGTCGCACGAG | CATGGAGGCCTCGTATGTG | 370 |
| HSA-LPL | TAGCAGAGTCCGTGGCTACC | TGGCACCCAACTCTCATACA | 410 |
| HSA-LDLR | GAGGTGGCCAGCAATAGAAT | TCTCTGCTGATGACGGTGTC | 411 |
| HSA-MEF2A | GTGTCTGTGACAACCCCAAG | GAAGCCTTGAAGGGCTGAC | 329, 494 |
| HSA-VEGFA | GTGTGTGCCCACTGAGGAGT | TGTTGTGCTGTAGGAAGCTCA | 127, 494 |
| HSA-PPP2R2A | TGGTTACCCCAGAAAAATGC | ACCCTTCTGGTCTTTTGTCC | 136 |
| HSA-IRS1 | ATACTCGAGTGACCTCAGCAAATCCTCTTC | ATAGCGGCCGCATACCTCCATCCCACATCCA | 487b |
| HSA-CCL2 | TCTGTGCCTGCTGCTCATAG | CGAGCCTCTGCACTGAGAT | 495 |
| HSA-IκBα | TTTTGGTGTCCTTGGGTGCT | CAACAGGAGTGACACCAGGT | 300 |
| HSA-BRD7-FW | CTGTTGCACTCAGGAATGAAAA | TCTGCTTTCGAGTTTTCTGCA | 300, 410 |
| HSA-FOXO1-FW | AGTGGATGGTCAAGAGCGTG | GCACACGAATGAACTTGCTGT | 544 |
| HSA-STAT3-FW | GTCAGTGACCAGGCAGAAGA | CACGTACTCCATCGCTGACA | 544 |
| HSA-PAK1-FW | TCGAACCAGGTCATTCACAGA | GCTCTGGGGTTATCTGTGCA | 485-5p |
| HSA-EMMPRIN-FW | GTACTCCTGCGTCTTCCTCC | CCCCTCGTTGATGTGTTCTGA | 485-5p |
| HSA-DNMT1 | ATCTTCCTGACACCCTGCAT | CTCCCTGGTAGAATGCCTGA |  |
| HSA-DNMT3A | AAGGAGGAGCGCCAAGAG | ATCACCGCAGGGTCCTTT |  |
| HSA-DNDMT3B | ATGAAGGTTGGCGACAAGAG | CCCTGTGAGCAGCAGAAACT |  |
| Pri-miR-329-1 | TGGGGAAGAATCAGTGGTGT | GACCAGAAGGCCTCCAAGAT |  |
| Pri-miR-329-2 | TGTCAAGTTTGGGGAAGGAA | GACCAGAAGGCCTCCAAGAT |  |
| Pri-miR-487b | AGGCAGTGGCTTTCTTTTCC | GAGGTGGGATCCAAACACAG |  |
| Pri-miR-494 | GATTCGGCAGTTCTGTTTTGA | CTGAAGGCTGCATCAGGAAC |  |
| Pri-miR-495 | CTGACCCTCAGTGTCCCTTC | ATGGAGGCACTTCAAGGAGA |  |

**Supplementary Figure 1: Correlations between DNA methylation and DNMT gene expression.** Vessels were divided into the following groups: lower limb arteries from patients with PAD (N=11), arteriae mammariae from patients with CAD (used as arterial graft) (N=18), VSMs from patients with CAD (used as vein graft) (N=8), lower limb veins from patients with PAD (N=10), lower limb veins from patients with critical ischemia (PAD-CI) (N=6). Linear regression analyses only showed statistically significant correlation between DNMT3A and MEG3-DMR-1A (p=0.03).

**Supplementary Figure 2. A. Peri-malignancy vessel versus non-malignancy vessel microRNA expression**. Peri-malignant vessels (N=19) versus non-malignant vessels (N=20) did not show significant differences for any of the analysed microRNAs. Mean expression per group is shown. The error bars represent the SEMs. MicroRNAs are arranged in the order of chromosomal location of the microRNA gene. **B. Peri-malignancy vessel versus non-malignancy vessel DNA methylation**. Peri-malignant vessels (N=13) versus non-malignant vessels (N=23) did not show significant differences for any of the analysed CpG sites. Mean expression per group is shown. The error bars represent the SEMs. CpG sites are arranged in the order of chromosomal location.
