# Supplementary figures and images for: miRMap: Profiling 14q32 microRNA Expression and DNA Methylation Throughout the Human Vasculature
Source: Front Cardiovasc Med. 2019 Aug 8;6:113. doi: 10.3389/fcvm.2019.00113 (PMC6694280; doi:10.3389/fcvm.2019.00113)

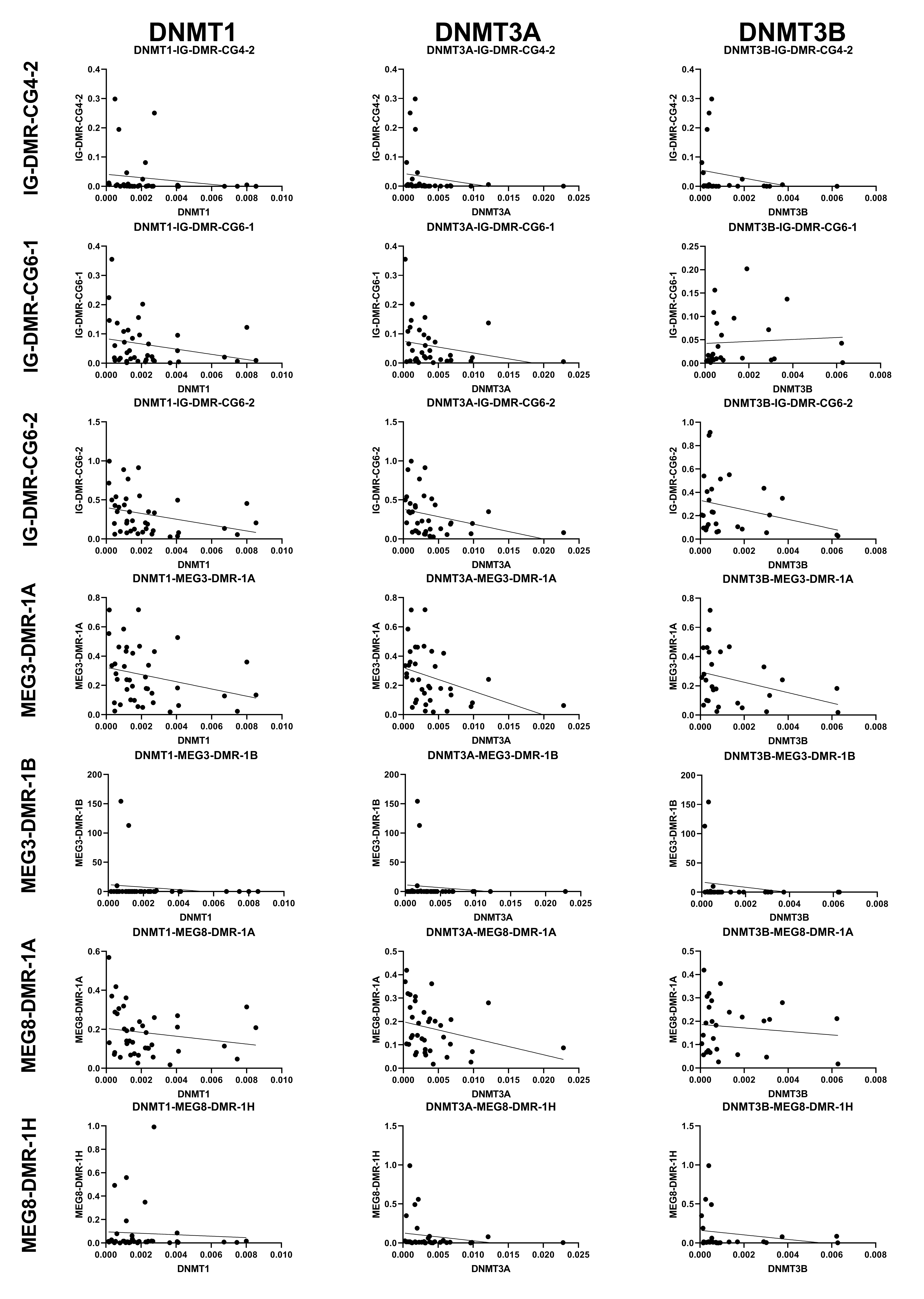

Supplement: Supplementary file 2 [file Image_1.tif]

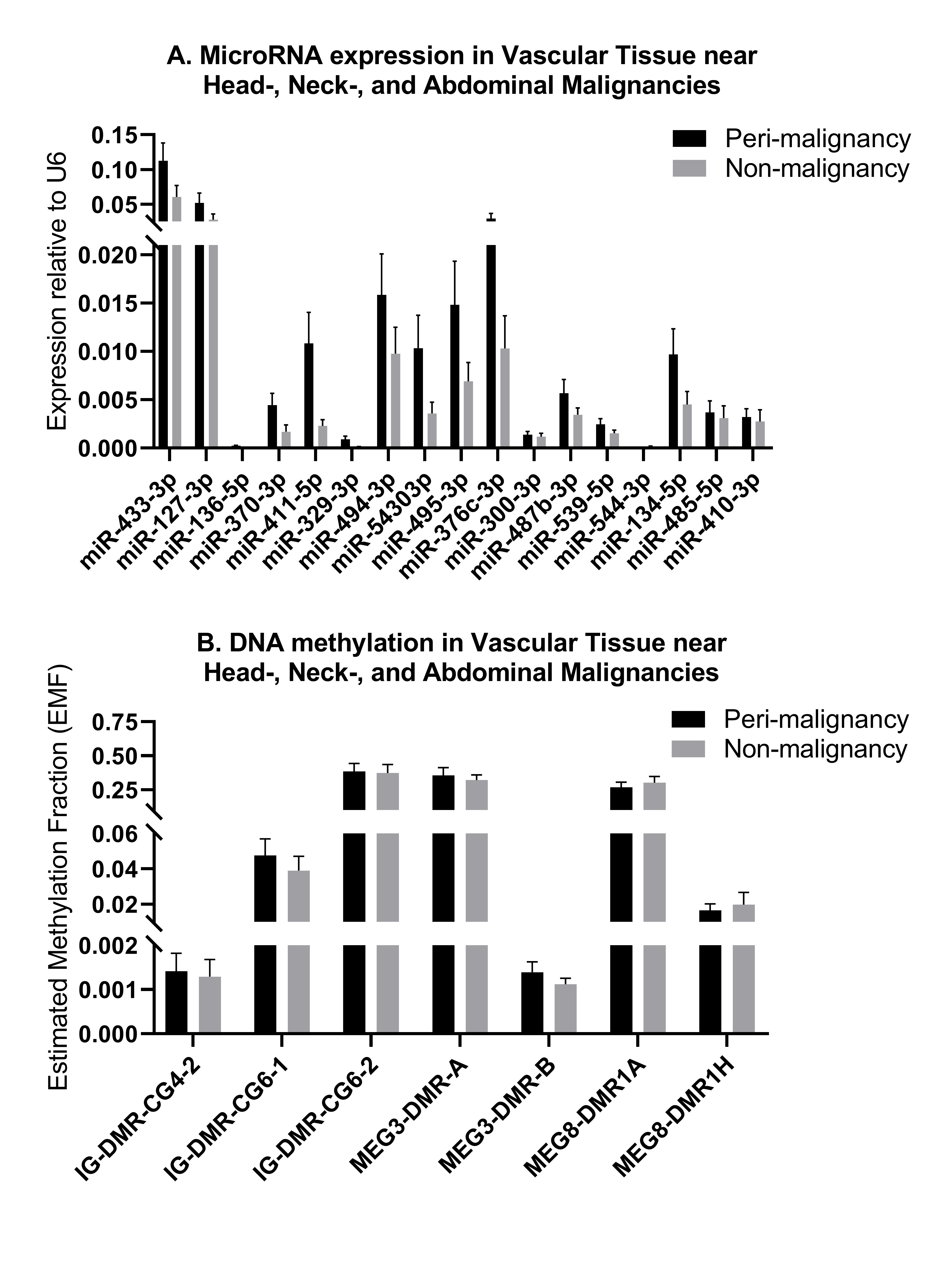

Supplement: Supplementary file 3 [file Image_2.tif]
